# The Role of γδ T Cells as a Line of Defense in Viral Infections after Allogeneic Stem Cell Transplantation: Opportunities and Challenges

**DOI:** 10.3390/v14010117

**Published:** 2022-01-10

**Authors:** Anke Janssen, Eline van Diest, Anna Vyborova, Lenneke Schrier, Anke Bruns, Zsolt Sebestyen, Trudy Straetemans, Moniek de Witte, Jürgen Kuball

**Affiliations:** 1 Department of Hematology, University Medical Center Utrecht, 3584 CX Utrecht, The Netherlands; a.janssen-7@umcutrecht.nl (A.J.); G.C.M.Straetemans@umcutrecht.nl (T.S.); M.A.deWitte-7@umcutrecht.nl (M.d.W.); 2 Center for Translational Immunology, University Medical Center Utrecht, 3584 CX Utrecht, The Netherlands; E.vanDiest@umcutrecht.nl (E.v.D.); A.Vyborova-2@umcutrecht.nl (A.V.); L.Schrier-3@umcutrecht.nl (L.S.); Z.Sebestyen@umcutrecht.nl (Z.S.); 3 Princess Maxima Center for Pediatric Oncology, 3584 CX Utrecht, The Netherlands; 4 Department of Infectious Diseases, University Medical Center Utrecht, 3584 CX Utrecht, The Netherlands; A.H.W.Bruns@umcutrecht.nl

**Keywords:** γδ T cells, allogeneic stem cell transplantation, viral infections, CMV, EBV, T cell depletion

## Abstract

In the complex interplay between inflammation and graft-versus-host disease (GVHD) after allogeneic stem cell transplantation (allo-HSCT), viral reactivations are often observed and cause substantial morbidity and mortality. As toxicity after allo-HSCT within the context of viral reactivations is mainly driven by αβ T cells, we describe that by delaying αβ T cell reconstitution through defined transplantation techniques, we can harvest the full potential of early reconstituting γδ T cells to control viral reactivations. We summarize evidence of how the γδ T cell repertoire is shaped by CMV and EBV reactivations after allo-HSCT, and their potential role in controlling the most important, but not all, viral reactivations. As most γδ T cells recognize their targets in an MHC-independent manner, γδ T cells not only have the potential to control viral reactivations but also to impact the underlying hematological malignancies. We also highlight the recently re-discovered ability to recognize classical HLA-molecules through a γδ T cell receptor, which also surprisingly do not associate with GVHD. Finally, we discuss the therapeutic potential of γδ T cells and their receptors within and outside the context of allo-HSCT, as well as the opportunities and challenges for developers and for payers.

## 1. Allogeneic Stem Cell Transplantation Platforms and Viral Reactivations

Allogeneic hematopoietic stem cell transplantation (allo-HSCT) is the only curative treatment for many hematological malignancies and non-malignant diseases in adults and children. In hematological malignancies, durable remission after this form of immunotherapy depends on the desired graft-versus-leukemia effect but it comes at a cost. Treatment related mortality can be as high as 30% mainly because of graft-versus-host-disease (GVHD) and infectious complications. Many different allo-HSCT platforms are currently used to counterbalance these risks and overall these achieve an improved GVHD-free overall survival (reviewed in [1]). To date, the majority of allo-HSCT platforms are based on the principle of T cell depletion. T cell depletion techniques include in vivo T cell depletion through anti-thymocyte globulin (ATG) [2,3,4,5], alemtuzumab [6,7], post-transplantation cyclophosphamide [8,9,10], and ex vivo graft engineering by CD34^pos^ cell selection [11,12] or αβ T cell depletion [13,14,15,16] using a recently well-described anti-αβ T cell receptor (TCR) antibody [17] (Table 1). The main challenge in choosing between platforms is the lack of standardized outcome reports. In addition, substantial variations in patient characteristics, as well as in type of disease and remission status, further hamper valid comparison. Comparing the incidence of viral reactivations or infections between different platforms is even more challenging. The viral infections most frequently seen after allo-HSCT are cytomegalovirus (CMV) and Epstein-Barr virus (EBV), herpes virus 6 (HHV-6), BK pyelomavirus (BKV), and adenovirus (ADV) [18]. In addition to the rather scattered reporting on viral reactivations in different studies (Table 1), a lack of reporting on frequency of, e.g., reactivation in relation to patients at risk and the use of different prophylactic and pre-emptive viral detection and treatment strategies hamper proper analyses. Despite the lack of detailed reporting on viral reactivations in most published studies, viral reactivations such as CMV reactivation were historically considered to be a major driver of increased morbidity and mortality [19]. Infections are a driver for inflammation and inflammation in the presence of αβ T cells is a driver for GVHD [19,20]. Another cause for inflammation is the classical myeloablative chemotherapy given prior to the infusion of stem cells [19,20,21,22], though newer reduced toxicity myeloablative regimens, such as Busulfan, given intravenously in combination with drug monitoring reduces such risks [23,24]. The use of unrelated donors for allo-HSCT has also been linked to viral reactivations, as these were often used in combination with T cell depletion strategies. However, as most centers currently also use T cell depletion with family donors, the observed increase in viral reactivations after the use of grafts from unrelated donors compared to related donors might not appear in future studies. The most important observation to date is that early immune reconstitution is associated with positive clinical outcomes [25,26,27,28,29,30]. This emphasizes that the true driver of toxicities after allo-HSCT is the amount of inflammation at a certain time point after allo-HSCT within the context of defined immune repertoires at the moment of inflammation [31,32,33].

## 2. A Common Immunological Nominator for All Transplantation Platforms

Various transplantation platforms differ substantially in immune reconstitution, although lack of harmonization in reporting and time points of the analyses make comparisons between platforms challenging [1]. In general, NK cells and γδ T cells recover within the first weeks after αβ T depleted allo-HSCT [13,34], whereas ATG-based platforms hamper the reconstitution of αβ T cells [35]. This is evidenced by the clinical observation that GVHD is rather high when T cell depletion is not performed. The rates of GVHD drop substantially after ATG treatment [2,3,4,5], though the level of GVHD might heavily depend on the administered dose [33,35,36]. Even lower incidences of, in particular, chronic GVHD are seen after post-transplantation cyclophosphamide [8,9,10] and ex vivo graft engineering through αβ T cell depletion [13,14,15,16]. One common nominator of success for all platforms seems to be the recovery of the numbers of CD4+ αβ T cells after allo-HSCT, although recovery is slow and can take months or even years [37]. Although slowly repopulating, numbers of CD4+ αβ T cells early after allo-HSCT have been reported as a major predictor for viral reactivations and improved clinical outcome [25,26,27,28,29,30,38]. Recovery of innate immune cells, defined as neutrophil, monocyte, and NK cells, have been proposed as a good predictor for CD4+ αβ T cell reconstitution [29]. NK and γδ T cells are part of the first innate immune cells to reconstitute after allo-HSCT [13,39,40], though their clinical impact has not been thoroughly investigated yet.

## 3. γδ T Cells, the Frequently Forgotten Child, within the Context of Allo-HSCT

γδ T cell subsets exhibit distinct developmental properties, tissue localizations, and activation modes. Generally, human γδ T cells are divided into two major structural subsets according to their TCR δ chain usage: Vδ2^pos^ T cells and Vδ2^neg^ T cells. The majority of the Vδ2^neg^ T cells express the Vδ1 chain and co-express different Vγ chains, whereas the majority of Vδ2^pos^ T cells co-express Vγ9. In general, Vγ9Vδ2 T cells are the main subset of γδ T cells in the peripheral blood and are able to recognize infections such as tuberculosis and malignant cells [41,42]. The anti-viral capacities of γδ T cells have been described for different viruses such as CMV, EBV, influenza, and HCV (hepatitis C virus), and more recently SARS-CoV-2 [43]. γδ T cells act as early responders with the upregulation of Toll-like receptors (TLRs) which recognize pathogen-associated molecular patterns (PAMPs), such as viral particles, initiating a cascade which leads to the production of interferons and pro-inflammatory cytokines [44]. γδ T cells also express NK type receptors, such as natural killer group member 2-D (NKG2D) which are activated in response to stress of, for instance, virally infected cells and mediate the production of both perforins and granzyme B, thereby increasing cytotoxicity [45]. Finally, γδ T cells can be directly activated by their TCR upon viral infection and produce several cytokines of which IFN-γ is the best studied. Activated γδ T cells have a direct cytotoxic function by inducing apoptosis of virally infected cells but they also have an indirect effect by recruiting the immune system by producing pro-inflammatory cytokines [43].

Despite increasing interest in γδ T cells, the vast majority of studies on immunological immune repertoires after allo-HSCT do not include γδ T cells in their analyses, though γδ T cells comprise up to 10% of the peripheral T cells in healthy individuals [41]. The reasons for not adding γδ T cells to immune monitoring panels might be manifold. For example, detection of γδ T cells is technically difficult, as, e.g., anti-CD3 antibodies can block γδ TCR staining and an appropriate choice of antibody clones is essential. However, as γδ T cells have the potential to control viral infections and tumor cells, efforts should be made to overcome these barriers so that more can be learned about their role within the immune reconstitution after allo-HSCT.

Reconstitution of γδ T cells and its relation to clinical outcomes after allo-HSCT has not been studied extensively. Results of single-center studies suggest a favorable role of γδ T cells after allo-HSCT, where an increased number of γδ T cells after allo-HSCT is associated with improved relapse-free survival and overall survival [46,47,48,49]. One study, however, showed that increased numbers of CD8^pos^ γδ T cells in the graft, a minor subset of all γδ T cells, were associated with an increased cumulative incidence of acute GVHD [50]. This specific subset seems to be more prone to allo-reactivity and thereby GVHD, with the upregulation of activation markers after in vitro mixed lymphocyte reaction (MLR) when compared to CD8^neg^ γδ T cells [50]. The same study showed that an increase in CD27^pos^ γδ T cells, which are capable of producing IFN-γ, in the graft was correlated with less relapse [50]. Despite these scattered reports on γδ T cells in relation to clinical outcomes, a recent meta-analysis confirmed favorable outcomes for event-free survival and overall survival in patients with increased numbers of γδ T cells after allo-HSCT [51]. Moreover, higher numbers of γδ T cells were associated with fewer relapses and fewer viral infections [51]. No association with the number of γδ T cells and the occurrence of GVHD was observed [51]. This is consistent with the observation that transplantation techniques, which heavily depend on NK and γδ T cells, associate with low incidences of GVHD [1,13].

The underlying molecular mechanism used by γδ T cells to control hematological malignancies in the first months after allo-HSCT is based on the ability of γδ T cells to recognize their targets in a major histocompatibility complex (MHC)-independent manner. Therefore, γδ T cells do not cause substantial GVHD, in contrast to αβ T cells, while they still exert their effect on tumor cells and virally infected cells [52]. Vγ9Vδ2 T cells sense changes in phosphoantigens (pAg) via their TCR. The current working hypothesis is that pAg accumulation within the cell results in changes in BTN2 and BTN3, modulated by RhoB, which can be sensed by the Vγ9Vδ2 TCR [53,54,55,56,57,58,59]. Ligands for Vδ2^neg^ γδ T cells, the dominant population in tissues, are less thoroughly described [41,60]. An interesting unexpected feature of Vδ2^neg^ γδ T cells is that they have been reported to cross-recognize classical HLA molecules, such as HLA-A24, by their γδ TCR [61]. Surprisingly, for this particular Vγ5Vδ1 TCR, normal cells are not recognized most likely because clustering of the HLA-A24 molecule on the cell membrane is important for recognition, which might differ between healthy and tumor cells [61]. The cross-recognition of classical HLA molecules by selected γδ TCR could be interesting to explore for controlling underlying hematological malignancies across HLA barriers and for genetic engineering strategies [62,63,64]. This finding is neither an artifact nor a rare event and is supported by findings from more than two decades ago when others had already described HLA A24 reactive γδ T cells and γδ TCRs [65]. Additionally, HLA-A2 [66] and B27 [67]-specific γδ T cells have been described, though no detailed analyses to study cross-reactivity towards healthy tissues has been performed for these TCRs.

## 4. CMV Infections Alter the γδ T Cell Repertoire after Allo-HSCT

Repertoire studies of the γδ TCR after allo-HSCT showed that the diversity of the repertoire recovered within the first months after allo-HSCT and remained stable thereafter [40]. The γδ TCR repertoire after allo-HSCT seems to be mainly based on de novo generation of γδ T cells, although also γδ TCRs identified in the graft could be found in the patient’s new repertoire [40]. Viral reactivation after allo-HSCT, in particular after CMV reactivation, resulted in a skewed γδ TCR repertoire with an expansion of specific Vδ2^neg^ γδ T cell clones [40,68,69,70]. These clones usually have a Vδ1 TCR but expansions of Vδ3^pos^ T cells have also been described [40,69]. Both Vδ1^pos^ and Vδ3^pos^ T cells are more often found in epithelial tissue where viral replication takes places, which explains their increase after CMV infection [45,70]. The increase in Vδ2^neg^ γδ T cells after CMV infection was shown in different transplantation settings, such as in T cell replete, umbilical cord, and HLA-haploidentical transplantations [49,69,71,72,73,74]. These observations are also supported by γTCR chain (TRG) sequencing analyses, which imply that despite CMV infection reshaping the TRG repertoire, TRG composition is not associated with aGvHD development [75].

The killing capacity of polyclonal Vδ2^neg^ γδ T cells isolated from patients with a CMV reactivation was assessed by different laboratories and in vitro co-culture of those cells with CMV-infected fibroblasts showed specific lysis and interferon-γ production, as well as cross-reactivity against different tumors [69,74,76,77,78]. This provides a potential explanation for the paradox that CMV reactivation associates with improved leukemia control [79,80,81] mainly in T cell-depleted platforms. However, in T cell replete transplantation platforms, CMV seropositivity of the patient and/or donor is frequently associated with an increased non-relapse mortality, even with preemptive treatment programs for CMV after allo-HSCT [82,83]. This might be caused because, as has been observed, CMV reactivation within the context of T cell replete transplantation platforms leads to extensive inflammation and GVHD. In line with this, a large database study could not confirm the protective effect of CMV on relapse and even showed an increase in transplant-related mortality in patients with CMV reactivation [84] (for review [85]). This is in contrast to data from T cell-depleted allo-HSCT and CMV reactivations, which are more in line with the first reports on the γδ T cell response upon CMV infection in kidney transplanted patients, a clinical scenario where major inflammation is missing [86]. In this cohort, the expansion of γδ T cells was driven by Vδ2^neg^ γδ T cells and the oligoclonality of the γδ T cell receptor repertoire in the CMV-infected patients is suggestive for in vivo antigen-driven selection of Vδ2^neg^ γδ T cells [87]. The expansion of γδ T cells was associated with the resolution of CMV infection, which points to a protective role of γδ T cells in CMV [88]. In addition to the Vδ2^neg^ γδ T cells, the recent occurrence of a Vδ2-positive but Vγ9-negative γδ T cell population has been described in CMV infections after kidney transplantation. The expansion of this population is more outspoken in severe cases of CMV disease [89]. Whether such cells also play a role after allo-HSCT needs further investigation.

## 5. γδ T Cells Immune Reconstitution after Allo-HSCT and Interplay with EBV Infections

EBV reactivations are a common complication of allo-HSCT, though they are less frequently observed than CMV reactivations (Table 1). However, when not controlled, post-transplantation lymphoproliferative disease (PTLD) is a rare but feared complication of EBV reactivation, mainly observed after T cell-depleted transplantations prior to the era of anti-CD20 therapies [90]. Patients with low Vγ9Vδ2 T cell numbers after allo-HSCT from HLA-haploidentical donors have been reported to have increased incidences of EBV-reactions [91]. Interestingly, for the αβ T cell-depleted platform, EBV reactivations are quite frequent in the absence of CD19-depletion [13], while adding CD19-depletion substantially reduces EBV reactivations (M. de Witte, J. Kuball, unpublished observations). This finding is, on the one hand, surprising, as studies on the mode of action of the Vγ9Vδ2 T cell receptor were performed in EBV-transformed B cells and allowed us to identify RhoB as a key modulator for the recognition of tumor cells by a Vγ9Vδ2 TCR [58], implying that γδ T cells can control EBV reactivation. However, recognition of EBV-transformed B cells depended on the additional administration of aminobisphosphonates and was donor-dependent. Additionally, within one donor, the capability to recognize EBV-transformed B cells varies substantially [55]. Others have also shown that aminobisphosphonate pamidronate-expanded human Vγ9Vδ2 T cells efficiently kill EBV-transformed autologous lymphoblastoid B cell lines through Vγ9δ2TCR and NKG2D receptor triggering, as well as through Fas and TRAIL engagement [92]. Thus, the underlying mechanism of donor dependency is most likely mediated by genetic variations, which allow some patients to control EBV reactivations by Vγ9Vδ2 T cells, while others lack the ability to properly activate this pathway due to single-nucleotide polymorphism (SNPs) [58]. This observation is in line with a report showing that different individuals mount different types of innate immune responses after EBV exposure. While one type of immune response utilizes NK and Vγ9Vδ2 T cells during EBV reactivation, others are only able to expand NK cells [93].

Vδ1^pos^ γδ T cells likely also play a role in EBV infection, as the expansion of these cells was seen in primary EBV infection [94]. In the context of allo-HSCT, skewing of the γδ T cell receptor repertoire towards oligoclonal Vδ1^pos^ γδ T cells after EBV reactivation has been reported [68]. In this study, an in vitro expanded Vδ1^pos^ T cell clone showed cytotoxicity against EBV-LCL. EBV-infected cells could also induce in vitro oligoclonal expansions of autologous Vδ1^pos^ γδ T cells from EBV-seropositive individuals. Furthermore, after cord blood transplantation in a patient with a prolonged EBV reactivation, Vδ1^pos^ γδ T cells expanded, which showed lytic activity against EBV-LCL [95]. However, other studies did not report Vδ1^pos^-positive γδ T cell expansion after EBV reactivation [69].

## 6. The Role of γδ T Cells in Other Viruses after Allo-HSCT: An Unexplored Field

While human γδ T cell responses and their anti-viral capacities after allo-HSCT are best studied in herpes viruses such as CMV or EBV, studies regarding the role of γδ T cells in other herpes viruses such as HHV-6 and varicella-zoster virus (VZV), or non-herpes viruses such as ADV and BKV, are lacking. Data on γδ T cell responses to infections with other herpes viruses, although rare after allo-HSCT, is available only outside the context of allo-HSCT, but is informative on the role of γδ T cells in viral infections. For example, in kidney-transplanted patients, no increase in γδ T cells was observed after infection with other herpes viruses such as varicella zoster virus (VZV), herpes simplex virus (HSV), or EBV [86]. Reports on human herpes virus 8 (HHV-8) showed that upon infection, an increase in Vδ1^pos^ γδ T cells is observed [96]. Additionally, Vδ1^pos^ γδ T cell activation was observed when the PBMCs of infected patients with HHV-8 were stimulated with viral particles of HHV-8. In addition, Vδ1^pos^ γδ T cells could decrease the release of viral particles in HHV-8-infected cell lines. Interestingly, the γδ T cell response in herpes simplex virus (HSV) was reported to consist mainly of Vγ9Vδ2 T cells [97,98]. Based on our current knowledge, these Vγ9Vδ2 T cells probably did not recognize a specific viral antigen because in vitro experiments showed lysis by these Vγ9Vδ2 T cells of not only HSV-infected cells but also of cells infected with other viruses. γδ T cell responses in non-herpes viruses are, among others, studied in HIV, influenza, and recently SARS-CoV-2. In primary HIV infection, the depletion and loss of activation potential of Vδ2^pos^ γδ T cells was observed together with an increase in Vδ1^pos^ γδ T cells. In elite controllers, the Vδ1^pos^ γδ T cell expansion was even more pronounced, suggesting that they play a role in controlling the virus [99]. More recently, the severe depletion of Vδ2^pos^ γδ T cells together with an increased differentiation and activation profile has been described in severe SARS-CoV-2 infection [100,101]. Activated Vγ9Vδ2 T cells were capable of killing influenza-infected lung alveolar epithelial cells in vitro, showing the potential contribution to viral clearance at the actual site of the infection [102]. Taken together, viral infections consistently alter the composition and phenotype of the γδ T cell compartment, and the anti-viral capacity of γδ T cells has been demonstrated in vitro. However, the exact role of γδ T cells in viral disease and their contribution to viral clearance in relation to other immune cells remain to be elucidated.

## 7. Unmodified γδ T Cells for Treatment of Viruses after Allo-SCT

To improve immune reconstitution and enhance the graft-versus-leukemia effect after allo-HSCT, different variants of donor lymphocyte infusion (DLI) have been studied. DLIs are administered as a prophylactic, pre-emptive, or therapeutic treatment; consist of either unmanipulated or manipulated cell products; and have additional value within the context of T cell-depleted allo-HSCT [1]. Consensus regarding the timing and dosing of DLI is lacking and currently primarily depend on the allo-HSCT platform (reviewed in [31]). For pragmatic reasons, DLIs are not purified and are thus mainly comprised of αβ T cells, however they also harbor many other immune subsets, including NK and γδ T cells. However, when analyzing the mode of action of unmanipulated DLI, the main focus is usually on αβ T cells. Additionally, only a limited number of reports are available on either the modulation of γδ T cells by drugs or on the infusion of isolated γδ T cells. Infusion of predominantly NK and γδ T cells with αβ T cell-depleted grafts during allo-HSCT and observed incidences of CMV and EBV reactivation when compared to T-cell replete allo-HSCT imply a strong ability of γδ T cells to control CMV reactivation. However, the capacity to control EBV reactivations seems to be limited in the absence of phosphoantigen-stimulating agents (Table 1). Aminobisphosphonates, such as pamidronate or zoledronic acid, are phosphoantigen-stimulating drugs and have been used extensively, sometimes in combination with interleukin-2 (IL-2) to stimulate Vγ9Vδ2 T cells in vitro. Aminobisphosphonates have few side effects and could, in theory, be used as a therapeutic tool after allo-HSCT to enhance the potential of Vγ9Vδ2 T cells to attack the underlying hematological malignancy, as well as EBV reactivations. In vivo or in vitro stimulation of autologous γδ T cells with aminobisphosphonates and/or IL-2 has been mainly studied in trials for cancer treatment, although no severe toxicity was reported and there was a lack clinical efficacy [41,42]. A study which explored in vivo treatment with zolendronic acid in pediatric patients treated with αβ T cell-depleted allo-HSCT reported no severe toxicities but patient numbers were too small to assess the impact on viral infections or relapse [73]. In vivo treatment with zolendronic acid, however, did lead to Vδ2^pos^ γδ T cell differentiation with increased cytotoxicity against leukemic blasts in vitro. Interestingly, the percentage of the Vδ1^pos^ γδ T cells of patients treated with zolendric acid was increased and also these Vδ1^pos^ γδ T cells showed increased cytotoxicity against leukemic blasts. There is no explanation for this unexpected finding but the authors speculate about the role of the bloom syndrome protein (BLM), which was found to be upregulated in γδ T cells treated with zolendronic acid [73]. BLM is involved in the development and maintenance of αβ T cells [103]. These findings exemplify the gaps in knowledge about the complex interplay between Vδ2 and both Vδ1 γδ T cells and αβ T cells.

## 8. Picking and Engineering Winners from γδ T Cells and Their Receptors for Future Anti-Viral Therapies

The most recent insights further stress the inter and intra-individual diversity of Vγ9Vδ2 T cells, as very detailed clonal analyses imply that many high frequency Vγ9Vδ2 T cell clones are poorly active against EBV-transformed or solid cancers [55]. Surprisingly, the NKG2A-positive subpopulation of Vγ9Vδ2 T cells is a source for more active clones, though optimal responses are observed with additional blocking through anti-HLA-E-interfering agents [104]. These data would suggest that the selection of NKG2A-positive subpopulations of Vδ2^pos^ T cells before infusion or HLA-E-blocking antibodies should be explored for future Vγ9Vδ2 T cell therapies. To harvest the potential of Vδ2^neg^ γδ T cells, the expansion of polyclonal Vδ1^pos^ γδ T cells, the so-called Delta One T cells (DOT), has been proposed and is currently being tested in clinical trials [105,106]. Though such strategies are mainly being explored within the context of cancer treatment (for review [41,42]), they are also an interesting treatment option for viral reactivations. γδ T cell-based therapies would allow for overcoming limitations of, e.g., HLA-restricted off-the-shelf virus-specific T cell banks [107]. Additionally, using γδ T cells as carriers for virus-specific αβTCR was explored with CMV and ADV-specific αβTCR [108,109]. γδ T cells engineered with a chimeric antigen receptor (CAR) are currently under investigation but again in the context of cancer treatment [110]. Though the use of γδ T cells as a third-party carrier would avoid the risks of GVHD, the limited in vitro proliferation capacity of γδ T cells could hamper such a strategy.

**Figure 1:**
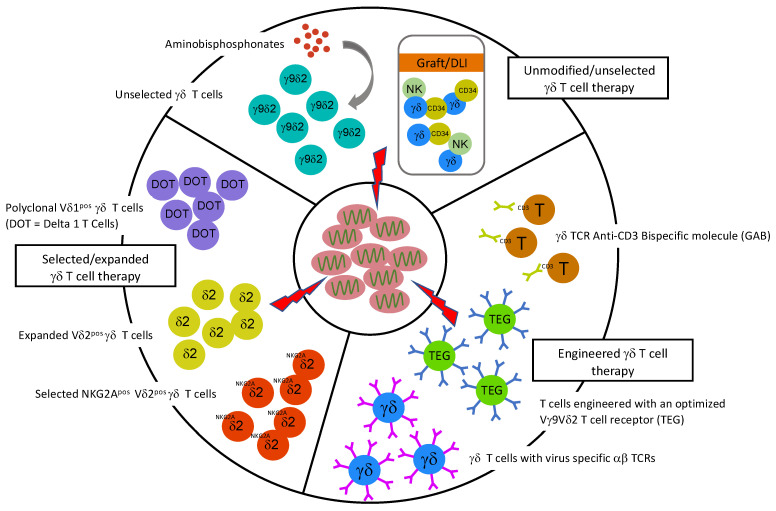
Possible γδ T cell-mediated therapies in viral infections.

Lastly, using highly active compounds to target infected cells could rely on engineering strategies. These can include either extracting tumor and virus reactive receptors, e.g., of Vγ9Vδ2 T cell receptors for the generation of T cells engineered with an optimized Vγ9Vδ2 T cell receptor (TEG) [55,58,64,111,112,113,114] or of Vδ2-negative γδ T cell receptors [60,61,62,64]. Such strategies allow for the generation of autologous long-lasting effector cells and overcome the HLA-restriction of engineered virus-specific αβ T cells. Finally, the so- called Vγ9Vδ2 T cell receptors have been used to generate a bispecific format [115], namely the so-called Gamma delta TCR Anti-CD3 Bispecific molecules (GABs), as novel immunotherapeutic compounds which could, e.g., in combination with aminobisphosphonates, allow for rapid off-the-shelf treatment for EBV reactivations or EBV-transformed lymphoma, and would also not depend on HLA-restriction. For an overview of possible γδ T cell-mediated anti-viral therapies, see Figure 1.

## 9. Conclusion within the Context of Other Anti-Viral Compounds and Re-Imbursement Dilemmas

To conclude, γδ T cells are currently often overlooked in studies regarding immune reconstitution and reports on both γδ T cells and viral infections are scarce. However, with their anti-viral capacities, they are believed to be an important line of defense in the inflammatory environment in the first months after allo-HSCT without causing GVHD. These beneficial properties have led to the development of allo-HSCT platforms where γδ T cells are an important pillar in the immune reconstitution. Moreover, γδ T cells are an interesting candidate for future cellular antiviral therapies after allo-HSCT. However, within the context of allo-HSCT, from a drug development perspective, major attention should also be drawn to other developed antiviral compounds, such as letermovir [116], which has been recently approved and is reimbursed in many countries for preventing CMV reactivation. A major assumption for prevention, as well as for treatment strategies, relies on an immune system which also harbors virus-reactive immune cells. Without these cells, only temporary control of the viral load can be achieved. Therefore, developing additional cellular immune therapies to enrich a fragmented immune repertoire remains a major field of interest. However, as allo-HSCT is already a costly intervention and access to it is not equal for all European citizens [117], additional high-end prices will most likely not be accepted by many payers. To date, overpriced products, in combination with a long production time, have only been accepted for CAR T cells because of their nature as a single intervention for a cure with a big impact [118,119]. After two decades of development, production time and pricing are likely the critical factors contributing to the failure of bringing other advanced cellular therapy products (ATMPs) to market, such as HSV-TK (thymidine kinase)-modified T cells [120], which were designed as add-ons to an allo-HSCT. The community that is working to develop such novel interventions should learn from these past failures and find ways to enable timely and affordable access to the market.

## Figures and Tables

**Table 1:** Studies reporting on type of transplantation and viral reactivations or infections. Adapted and modified from de Witte et al. [1].

Study	Patients	Donor	Intervention	Numbers	CMV	EBV	BK	Adeno
ATG								
Chang et al. [2]	Adult hematological malignancies	MRD	ATG-T	263	Day 100: 22.7%	Day 180: 7.8%	n.a.	n.a.
Walker et al. [3]	Adult hematological malignancies	MUD MMUD	ATG-T	101	n.a.	20% DNAemia requiring therapy	n.a.	n.a.
Finke et al. [4]	Adult hematological malignancies	MRD MUD	ATG-F	103	53.8% DNAemia 5.7% CMV disease	5% PTLD	n.a.	n.a.
Soiffer et al. [5]	Adult AML, MDS, and ALL	MUD	ATG-F	126	62% (R+) DNAemie	1.6% PTLD	n.a.	n.a.
**Alemtuzumab**								
Green et al. [6]	Adult hematological malignancies	Matched Mismatched	Alemtuzumab	313	>80% (R+) DNAemia	n.a.	n.a.	n.a.
Carpenter et al. [7]	Adult hematological malignancies	MRD MMRD MUD MMUD	Alemtuzumab	111	n.a.	2Y 40.3% DNAemia 1% PTLD	n.a.	n.a.
**PTCy**								
Cieri et al. [8]	Adult high risk hematological malignancy	Haplo	PTCy	40	63% DNAemia 17% CMV disease	15% DNAemia (66% of these pts treated). No PTLD	18%	n.a.
Berger et al. [9]	Pediatric; high risk hematological malignancy	Haplo	PTCy	33	36% DNAemia No CMV disease	3% DNAemia No PTLD	17%	3% DNAemia; Not symptomatic
Retiere et al. [10]	Adult hematological malignancies	MRD MUD MMUD haplo	PTCy vs. ATG-T	45	DNAemia PTCY 27% ATG 40%	DNAemia requiring treatment PTCY 0% ATG 33%	PTCY 3%ATG 0%	PTCY 15% ATG 20%
**αβT cell depletion**								
De Witte et al. [13]	Adult hematological malignancies	MRD MUD MMUD	αβT cell depletion	35	64% (R+) DNAemia 6% CMV disease	44%	n.a.	n.a.
Laberko et al. [14]	Pediatric malignant + non-malignant	MUD haplo	αβT cell/CD19 depletion	182	51%	33%	n.a.	n.a.
Maschan et al. [15]	Pediatric high-risk AML	MUD MMUD Haplo	αβT cell/CD19 depletion	33	52% DNAemia 6% CMV disease	50% DNAemia; 6% Rituximab	n.a.	n.a.
Bertaina et al. [16]	Pediatric non-malignant	Haplo	αβT cell/CD19 depletion	23	38% DNAemia CMV/adeno	50% DNAemia; 6% Rituximab	n.a.	38% DNAemia CMV/adeno

Abbreviations: Adeno = adenovirus; ALL = acute lymphoblastic leukemia; AML = acute myeloid leukemia; ATG = anti-thymocyte globulin; ATG-F = anti-thymocyte globulin-fresenius; ATG-T = anti-thymocyte globulin-thymoglobulin; BK = BK virus; CMV = cytomegalovirus; EBV = Epstein–Barr virus; haplo = haploidentical donor; MDS = myelodysplastic syndrome; MMRD = mismatched related donor; MMUD = mismatched unrelated donor; MRD = matched related donor; MUD = matched unrelated donor; NA = not available; PTCY = post-transplantation cyclophosphamide; PTLD = post-transplant lymphoproliferative disease; pts = patients; R+ = cytomegalovirus positive recipient; and y = year.
